# Growth rates and the prevalence and progression of scoliosis in short-statured children on Australian growth hormone treatment programmes

**DOI:** 10.1186/1748-7161-2-3

**Published:** 2007-02-22

**Authors:** Gregory A Day, Ian Bruce McPhee, Jenny Batch, Francis H Tomlinson

**Affiliations:** 1 Department of Surgery, University of Queensland, Brisbane, Australia; 2 Department of Paediatrics and Child Health, University of Queensland, Brisbane, Australia; 3 Level 5, St Andrews Place, 33 North Street, Spring Hill, Queensland, Australia 4000

## Abstract

**Study design and aim:**

This was a longitudinal chart review of a diverse group (cohort) of patients undergoing HGH (Human Growth Hormone) treatment. Clinical and radiological examinations were performed with the aim to identify the presence and progression of scoliosis.

**Methods and cohort:**

185 patients were recruited and a database incorporating the age at commencement, dose and frequency of growth hormone treatment and growth charts was compiled from their Medical Records. The presence of any known syndrome and the clinical presence of scoliosis were included for analysis. Subsequently, skeletally immature patients identified with scoliosis were followed up over a period of a minimum four years and the radiologic type, progression and severity (Cobb angle) of scoliosis were recorded.

**Results:**

Four (3.6%) of the 109 with idiopathic short stature or hormone deficiency had idiopathic scoliosis (within normal limits for a control population) and scoliosis progression was not prospectively observed. 13 (28.8%) of 45 with Turner syndrome had scoliosis radiologically similar to idiopathic scoliosis. 11 (48%) of 23 with varying syndromes, had scoliosis. In the entire cohort, the growth rates of those with and without scoliosis were not statistically different and HGH treatment was not ceased because of progression of scoliosis.

**Conclusion:**

In this study, there was no evidence of HGH treatment being responsible for progression of scoliosis in a small number of non-syndromic patients (four). An incidental finding was that scoliosis, similar to the idiopathic type, appears to be more prevalent in Turner syndrome than previously believed.

## Background

Research indicating that juveniles who develop idiopathic scoliosis have a markedly elevated growth rate compared to controls fostered a popular belief that growth hormone influences the progression of scoliosis[1,2]. Serum levels of growth hormone in children with idiopathic scoliosis were found to be elevated between the ages of seven and twelve years compared to controls [3-7]. Progression of structural scoliosis was reported in a short-statured patient during Human Growth Hormone (HGH) treatment [8] and stimulated a systematic clinical audit of children with scoliosis on an HGH treatment programme in one institution [9]. The administration of HGH appeared to accelerate the progression of scoliosis in six of ten children on that programme (cohort size 250) [9]. Of the ten, five had idiopathic scoliosis, one had acute lymphocytic leukaemia and two with Turner syndrome were diagnosed with scoliosis prior to treatment. An accelerated growth rate was also recorded in eight of ten with scoliosis.

Armed with this information, it was hypothesized that children with scoliosis, who are administered Human Growth Hormone, are more likely to have progression of their scoliosis and an accelerated growth rate compared to those without scoliosis. The aims of this study are to determine the prevalence of scoliosis in four Australian Institutions' Growth Hormone Treatment Programmes and to confirm the results of previous research that HGH treatment can be associated with progression of pre-existing scoliosis.

## Methods and subjects

### Study group

298 medical records were available for scrutiny and 30 were excluded for reasons outlined in *Exclusion criteria*. A database was completed for 185 (69%) of the remaining 268 patients. 83 (31%) refused to complete the mailed-out questionnaire and sign consent. Of the 185 patients, 97 had idiopathic short stature and 12 had either growth hormone or multiple pituitary hormone deficiency. 68 had a condition/syndrome known to be associated with scoliosis (Table 1). Eight patients had conditions not usually associated with scoliosis (Table 2).

**Table 1:** Syndromes/conditions known to be associated with scoliosis

**Diagnosis**	**Number of patients**	**Number with scoliosis**
Turner	45	14 (1 congenital)
Vater	2	2
Leri-Weill	2	2
Russell Silver dwarf	1	1
Klippel-Feil with Sprengel shoulder	1	1
Pierre-Robin	1	0
Stickler's	1	0
Noonan	1	0
Dubowitz	1	0
Neurofibromatosis	1	0
Cerebral Palsy	1	1
Spina Bifida	1	1
Multiple seizures	1	0
Cysteinosis	1	0
Fanconi Syndrome and Rickets	1	1
Pituitary Deep Xray therapy	1	1
Downs	1	1
Congenital rubella	1	0
Deep Xray therapy to the brain	4	0

**Table 2:** Conditions other than idiopathic short stature, not usually associated with scoliosis

**Diagnosis**	**Number of patients**
Renal failure	3
Polycystic kidney	1
Nephrotic syndrome	1
Glomerulo-nephritis	1
Arachnoid cyst	1
Gilbert's syndrome	1

### Exclusion criteria

Exclusion criteria for clinical examination included five children less than six years old and nineteen who commenced the *OZGROW *(The Australasian Paediatric Growth Hormone database) programme over the age of twelve years, because of their proximity to menarche. Two who signed Consent Forms and subsequently changed their minds were excluded. The Medical Records of four deceased were available for perusal, but were excluded from analysis.

### Database

The age at examination, gender, growth charts (Stature for Age – developed by the National Centre for Health Statistics in conjunction with the National Centre for Chronic Disease Prevention and Health Promotion 2000), the timing (age of commencement) and dosage and frequency of growth hormone treatment and the presence, site and severity of any detected spinal deformity were recorded for all who underwent clinical examination (Additional file 1). Turner syndrome growth charts were used specifically for that syndrome [10]. Turner syndrome karyotypes were recorded (Table 3). Information from the medical records revealed the presence of any known spinal deformity prior to the commencement of the HGH treatment.

**Table 3:** Turner syndrome scoliosis (non-congenital)

**Scoliosis**	**Karyotype**
12° Right thoracic, hypokyphosis	45XO
30° Right Thoraco-lumbar	45XO
18° Right thoracic, hypokyphosis	46XisoX(q10) Mosaic
30° Right Thoraco-lumbar	45XO
No Xrays Mild clinical deformity Right thoracic	45XO
17° Left Thoraco-lumbar	45XO
18° Right thoracic, hypokyphosis	45XO
55° Right thoracic Scoliosis surgery	45XO
45° Right thoracic, hypokyphosis	45XO
10° Right lumbar	Diagnosed South Africa. Karyotype not available.
20° Left Thoraco-lumbar	45XO
12° left Thoraco-lumbar	45XO/46XY mosaic Undiagnosed syndrome
45° Right Thoracic, hypokyphosis	45XO

### Growth chart information

Actual and percentile heights were recorded at three monthly intervals during the HGH treatment programme. Percentile, rather than actual, heights were included for analysis because the growth chart with actual heights for Turner syndrome has a different scale from the National Centre for Health Statistics scale.

### Clinical confirmation of spine deformity

Patients presented dressed in a white Hospital gown for a forward bend test (Adams), having removed their shoes [11]. One examiner (GD) used a scoliometer to measure for thoracic and lumbar spine rotation (Bunnell test) [12]. Patients with a straight spine and no truncal rotation were not asked to present for spine radiographs.

### Radiologic confirmation of the spine deformity

Spine radiographs of all patients with clinical signs of scoliosis have been performed with the exception of one with Turner syndrome and another who had undergone deep Xray therapy. Both were skeletally mature at the time of clinical examination. Standard postero-anterior and lateral complete spine radiographs in the erect posture were reviewed. Images at the time of clinical examination were in a hard-copy form and images after 2002 were in digital format. Cobb angles were drawn by the treating scoliosis surgeons and recorded.

### Measurement of progression of scoliosis

Two patients detected with previously unrecognised spine lateral deviation/trunk rotation were referred to the Scoliosis Clinic in their hospital, and subsequently underwent spine imaging every six-months to skeletal maturity. A third patient with previously unrecognised spine deformity was skeletally mature and underwent a single spine radiograph. Twenty-four with known scoliosis had undergone similar regular follow-up in Scoliosis Clinics, prior to clinical examination in this study (two continued follow-up after the 2002 clinical examination). Follow-up spine imaging ceased when patients became skeletally mature or Human Growth Hormone treatment ceased. An increase in Cobb angle of 10° was the minimum measurement used to define progression of scoliosis.

### Ethics

Institutional Ethics approval was granted from four hospitals in three Australian States to clinically examine all their patients treated through the OZGROW program.

### Statistical analysis

This study produced subgroups with data that could be analysed using a chi-square comparison (Student's t-test). Factors that might theoretically influence the presence or progression of scoliosis including the age of commencement of HGH treatment, the duration of therapy, the magnitude of each dose/average dose of growth hormone and the total amount of growth hormone administered to each patient were included for analysis. Step-wise multiple regression analysis used univariate models for each subgroup, where numbers for comparison exceeded 30 (Table 4).

**Table 4:** Statistical analysis for the cohort of 185. Predictors of Scoliosis: Logistic Regression – Univariate Models Results

	B	S.E.	Wald	Sig.	Exp(B)
Age Comm.	.039	.067	.335	.563	1.040
Duration	-.166	.099	2.814	.093	.847
Mg per Kg1	2.544	1.277	3.965	**.046**	12.728
Mg per Kg2	2.420	1.122	4.656	**.031**	11.245
Mg per Kg3	.716	2.085	.118	.731	2.046
Mg per Kg4	7.827	4.661	2.819	.093	2507.794
Last PCTL	-.015	.016	.863	.353	.985
Turner syndr.	-1.790	.471	14.430	**<.001**	.167
First PCTL_Age	-.046	.076	.364	.546	.955
First_PCTL	-.026	.024	1.255	.263	.974

B = Multiple regression co-efficient

S.E = Standard error

** Wald **(Wald Abraham. Selected papers in statistics and probability. Stanford University Press. New York, Toronto, London. 1955.)

Sig.= Significance

Exp (B) = Risk ratio

Each variable was used in turn to predict the occurrence of scoliosis in a logistic regression model. The size of the first and second doses of HGH (MG per KG1 and 2) and having Turner syndrome were predictive of having scoliosis. Variables for analysis where numbers exceeded 30 –

a) Age of commencement

b) Duration of HGH treatment

c) Dose of HGH in Mg/Kg body weight

d) First percentile height at commencement of HGH treatment (PCTL)

e) Last percentile height at cessation of HGH treatment (PCTL)

f) Presence of Turner syndrome

## Results

### Overall results

At review in 2001/2002, the mean age of the patients was 13.6 years (range 6–27 years). 91 were male and 94 were female. The mean age at commencement on the *OZGROW *programme was 8.5 years (range from birth to 12 years, and followed a normal distribution). The mean duration of HGH therapy up to the time of review was 4.7 years (range 2 months to 12 years). The findings from routine clinical and radiological follow-up for those in Scoliosis Clinics ceased in 2006, spanning 4–5 years duration (mean 4.6 years).

### Presence of scoliosis

29 of 185 patients had scoliosis. The duration of HGH therapy and total dose of human growth hormone were not related to the presence of scoliosis, although the average of the first two doses of growth hormone per patient, expressed in milligrams per kilogram body weight, was related to the presence of scoliosis. (p = 0.021). Having Turner syndrome was identified as the only highly significant factor for the presence of scoliosis (p = 0.001).

### Progression of scoliosis

The mean duration of HGH treatment for the entire cohort was 4.7 years (SD 2.8). A number of different HGH regimens were administered to the cohort including Saizen™, Norditropin™, Somaton™, Genotropin™ and Humatropin™. The mean dose of HGH treatment was 0.60 mg/Kg body weight (Range 0.25 – 1.14 mg/Kg body weight and SD 0.227). HGH therapy was ceased and restarted at a later stage in 13 children for reasons other than the presence of scoliosis. One male with Cushing's syndrome and mild thoracic scoliosis had a temporary three-month cessation of HGH treatment. HGH therapy was re-started and continued for another two years, with no radiologic progression of scoliosis. Progression of scoliosis was not recorded in two patients with idiopathic scoliosis and two with other conditions, followed up in Scoliosis Clinics between 2001/2 and 2006.

Progression of scoliosis had already occurred in five Turner and two 'other condition' patients at the time of clinical review in 2001/2002 (Cobb angles between 30° and 55°).

### Idiopathic short stature and hormone deficiency subgroup

Four (3.4%) of the 117 in this subgroup had scoliosis. All four had idiopathic right thoracic scoliosis less than 20 degrees Cobb angle. Three were male and all were unaware of their scoliosis until they were examined in this study. Two of the males had idiopathic short stature and the other had multiple pituitary hormone deficiency. Two of the three were skeletally mature and the other was age 13 at the time of clinical examination and continued review in the Scoliosis Clinic of his hospital for three years. The only female in this group had growth hormone deficiency and commenced HGH supplementation at age three years and remained on the *OZGROW *programme at age 13 years (in 2001). Both who continued clinical review demonstrated no radiologic progression of their scoliosis, from 2001/2 to 2006.

### Turner syndrome subgroup

**Figure 1:**
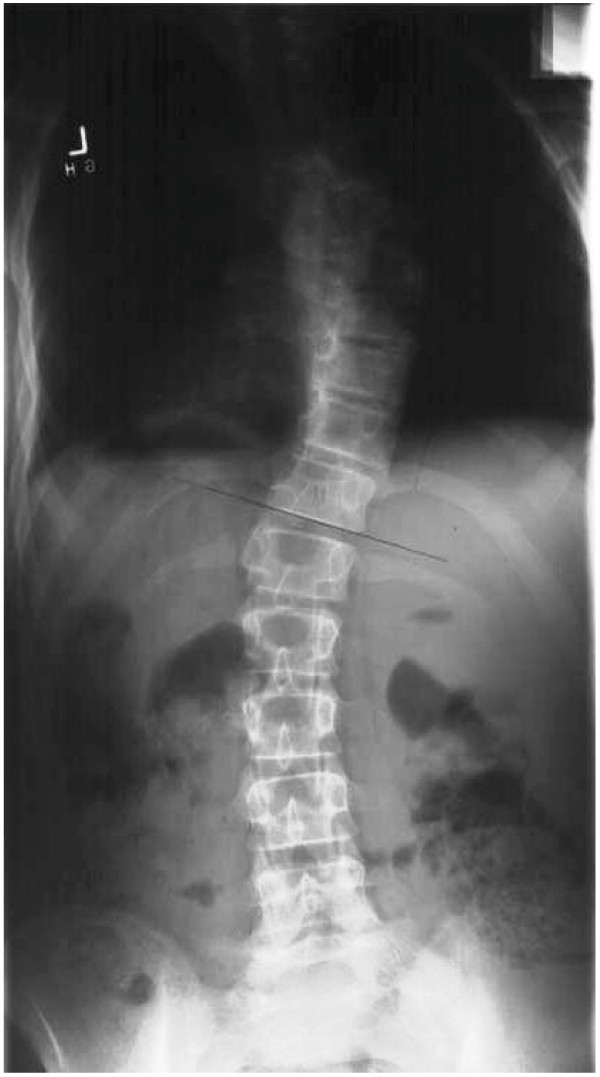
PA plain radiograph Turner syndrome scoliosis.

**Figure 2:**
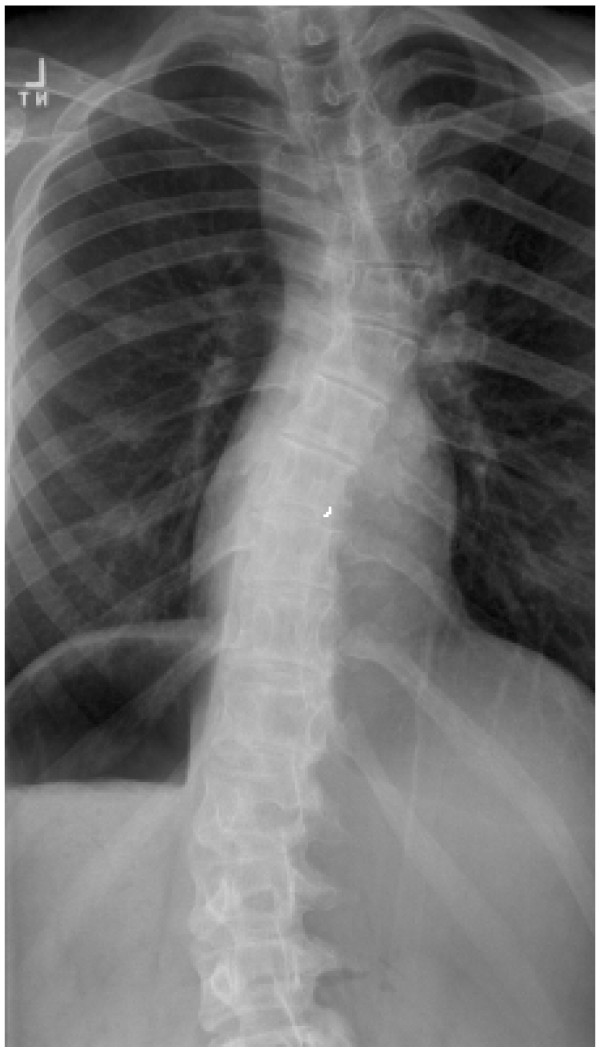
PA plain radiograph Turner syndrome scoliosis.

**Figure 3:**
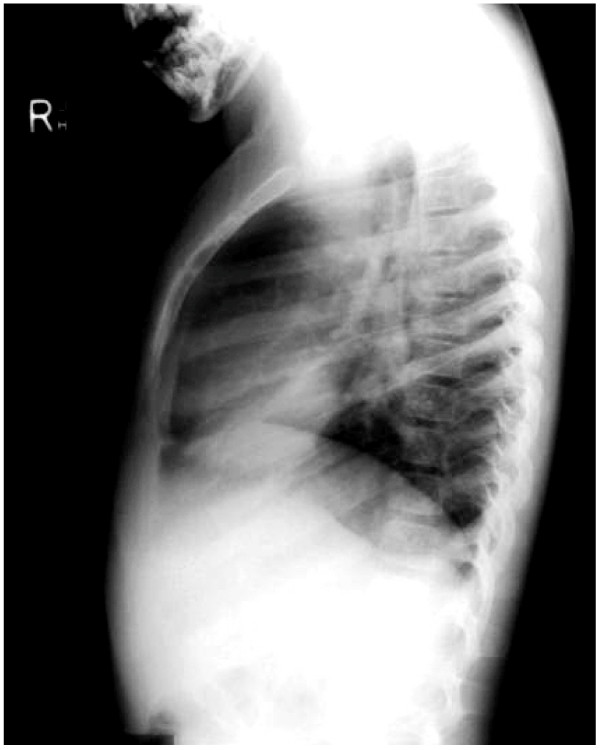
Lateral plain radiograph Turner syndrome scoliosis with hypokyphosis.

Of 45 with Turner syndrome, one had congenital scoliosis and underwent scoliosis surgery at age 13. Curve progression was not recorded for this patient. Thirteen of the 45 (28.8%) had scoliosis radiologically indistinguishable from idiopathic scoliosis (Figures 1, 2, 3). The mean age at diagnosis of scoliosis was 13 (range 3–22 years). All had oestrogen supplementation for delayed puberty. The site/morphology of the scoliosis and karyotype was recorded (Table 3). One had scoliosis prior to the commencement of HGH treatment and two others were diagnosed with scoliosis within one year of commencing the HGH treatment programme. The deformity subsequently progressed in both, resulting in right thoracic curves of 45°. Another underwent surgery for the scoliosis deformity, during HGH treatment. Analysis of the Turner syndrome subgroup revealed that they were administered a larger average dose of growth hormone (milligrams per kilogram body weight) (p = 0.003) and were commenced on the *OZGROW *program at an earlier age (p < 0.001) than the group comprising idiopathic short stature. The magnitude of the scoliosis curve in Turner syndrome was not influenced by the duration of HGH therapy.

### Other syndromes known to be associated with the presence of scoliosis

Scoliosis was present in 11 of the other 23 (48%) with varying other syndromes (Refer Table 1). One of this group had kypho-scoliosis.

### Growth velocity

There was a significant statistical correlation between the first and last percentiles of height in the idiopathic short stature and hormone deficiency subgroup (r = 0.514, p < 0.001) – ie. an acceleration of growth rate compared to peers was not statistically evident on the growth charts, between the commencement of the human growth hormone treatment programme and its cessation. The growth velocity of the four with scoliosis (mean 8.3 mm/year, range 7.0 to 10.6) in the idiopathic short stature and hormone deficiency subgroup was no different from the other 113 in the subgroup (mean 9.0 mm/year, standard deviation 7.53).

## Discussion

### Prevalence of scoliosis on the Human Growth Hormone treatment programme

In this study, the 97 patients with idiopathic short stature had a similar prevalence of idiopathic scoliosis to a control Caucasian population [13] and served as a 'control' group for comparisons with the other groups. The prevalence of scoliosis in this study was similar to previously published research, involving 31,462 patients on Human Growth Hormone treatment programmes [14-18]. Scoliosis is not a specific feature of idiopathic short stature and its prevalence should mirror that of the control population [19]. Although scoliosis has been extensively reported in specific syndromes associated with short stature, the commonest spinal deformity in short-stature syndromes and skeletal dysplasias is kyphosis [19].

### Prevalence of scoliosis in Turner syndrome on the HGH treatment programme

The 28.8% prevalence of scoliosis in Turner syndrome from this study was higher than previously published research, which includes those not treated with HGH (11–12%) [20-22]. In a previous HGH treatment study, a 'subgroup with Turner syndrome and those with a history of brain tumour or vertebral abnormalities had a higher prevalence of new and/or severe scoliosis than other groups'[15]. In a larger HGH treatment study, 72 of 24,000 children were diagnosed with scoliosis, of whom 10 had Turner syndrome [16].

### Progression of scoliosis on the Growth Hormone treatment programme

In this study, prospective follow-up from the four with scoliosis and idiopathic short stature/hormone deficiency indicated that there was no progression of the scoliosis during HGH treatment. The deformities in the current study were mainly single curve thoracic scoliosis and the results are similar to those of a previous study involving four single curves [9]. In the previous study, progression of scoliosis was associated with six double curves during HGH treatment, however it was not stated whether there was a temporary/permanent cessation of HGH treatment at the time of scoliosis progression [9].

In this study, retrospective data from the Turner syndrome patients' medical records indicated that the scoliosis deformity progressed in five of thirteen during HGH therapy and that the HGH treatment was not stopped because of this scoliosis progression.

### Growth velocity and progression of scoliosis

In this study, the growth velocity was similar in patients with and without idiopathic scoliosis. This result contrasts with that of a previous study, where accelerated growth was recorded in eight of ten with scoliosis compared to those without scoliosis [9].

## Conclusion

This study supports the contention that the administration of Human Growth Hormone to a diverse group of short-statured children does not lead to an increased prevalence of scoliosis. An accelerated growth rate during HGH treatment was not recorded in the small numbers with idiopathic scoliosis, contrasting with the results of the only other similar previous study. In this study, patients on HGH treatment with progressive scoliosis had syndromes in which the presence of scoliosis is commoner than an age-matched population.

A curious incidental finding was the 28.8% prevalence of scoliosis in Turner syndrome, which could mean that scoliosis is commoner in Turner syndrome than previously believed or that treating Turner syndrome girls with human growth hormone leads to an increased prevalence of scoliosis.

## Authors' contributions

This study was completed in the Institutions of the authors and those acknowledged. J B and F T were PhD Principle Advisors and I McP was Associate Advisor. All authors read, edited and approved the final manuscript.

## Supplementary Material

Additional file 1 Growth Rates and the Prevalence and Progression of Scoliosis in short-statured children on Australian Growth Hormone Treatment Programmes. The data provided represent the statistical analysis of doses of growth hormone, absolute and relative growth rates and the presence, progression and magnitude of scoliosis. Click here for file
