# Supplementary material for: Growth rates and the prevalence and progression of scoliosis in short-statured children on Australian growth hormone treatment programmes
Source: Scoliosis. 2007 Feb 22;2:3. doi: 10.1186/1748-7161-2-3 (PMC1808441; doi:10.1186/1748-7161-2-3)
Supplement: Additional file 1 — Growth Rates and the Prevalence and Progression of Scoliosis in short-statured children on Australian Growth Hormone Treatment Programmes. The data provided represent the statistical analysis of doses of growth hormone, absolute and relative growth rates and the presence, progression and magnitude of scoliosis. [file 1748-7161-2-3-S1.pdf]

Gender	Birthdate	GH start	age com	Duration	Extra	Syndrome	Deceased	Scoliosis
M	22.05.88	19.01.96	7.7			Nephrotic	Yes	N/A
F	10.06.78	10.01.88	9.5			Turner	No	Yes
F	26.03.91	22.05.98	7.1	4.5 years		Poly kidney	No	No
M	02.04.87	30.04.90	3	10 years		Panhypo	No	No
M	03.03.87	25.10.91	4.7	10.5 years		Hypokyph	No	No
F	26.09.83	09.11.90	7.1	3 years		Spina bifid	No	Yes
M	16.03.76	17.02.89	12.9	2 years		Seizures	No	No
F	09.02.79	10.06.88	9.5	6 years		ISS	No	Yes
M	05.09.87	02.11.98	12.1				No	No
M	02.09.80	30.10.92	12.1	4 years			No	No
M	05.05.82	12.06.97	15.1	2.5 years			No	No
M	05.10.77	31.05.91	13.5	4.5 years			No	No
F	05.04.80	20.08.91	11.3	6 years		pit tumour	No	No
M	05.05.88	09.01.95	6.5	5 years			No	No
F	26.11.79	09.03.92	12.3	"little"			No	No
M	30.01.76	01.06.90	14.4	3 years			No	No
M	17.05.89	22.01.96	6.7	7.5 years			No	No
F	28.07.83	07.06.90	6.9			Turner	No	No
F	07.03.84	02.12.91	7.7	8.5 years		Turner	No	No
M	14.03.86	02.08.91	5.4	5 years			No	No
F	14.11.91	08.12.97	6.1				No	No
F	15.05.92	03.06.96	4.1	7 years			No	No
F	21.12.81	11.01.93	11.1	6.5 years		Turner	No	No
M	19.03.81	17.03.89	8	5 years			No	No
F	13.06.82	16.07.93	11.1	5 years		?	No	No
F	27.04.83	02.08.96	13.3				No	No
F	20.12.82	13.10.95	12.9	4 years		Downs	No	Yes
M	03.04.81	19.10.92	11.5	3.5 years			No	No
F	29.12.80	03.08.90	9.7	5.5 years		Fanconi	No	Yes
						Rickets		
M	22.02.80	08.02.90	10	1 Year			No	No
F	19.05.79	25.11.88	9.5	5 years		Turner	No	Yes
F	07.02.88	26.08.96	8.5				No	No
M	03.04.79	14.05.93	14.1				No	No
M	Deceased						Yes	N/A
F	16.02.82	18.06.90	8.3	4 years		Turner	No	Yes
M	24.02.87	15.06.98	11.3	3.5 years		Addison's	No	No
F	19.08.84	08.07.96	11.9	2.5 years			No	No
M	20.04.80	12.08.88	8.3				No	No
F	14.04.80	25.11.88	8.6	4.5 years		Turner	No	No
F	10.04.87	16.02.98	10.9	5 years		Turner	No	Yes
M	24.03.86	07.12.92	6.8	8 years			No	No
M	26.12.74	16.09.89	14.7				No	Yes
M	Deceased	17.03.88					Yes	N/A
F	07.08.78	05.02.85	6.5	8 years			No	No
F	12.08.78	03.12.86	8.3	6 years			No	No
F	04.11.88	04.02.94	5.2	8.5 years	Madelung	Turner	No	No
M	01.02.86	04.12.98	12.8	3 years			No	No
F	12.09.85	11.05.98	12.7	3 years		Turner	No	Yes
F	08.10.81	29.11.88	7.1			Turner	No	Yes
M	27.02.86	28.08.92	6.5	9 Years			No	No

<20 RT	<30 RT	<40 RT	>40 RT	Cong Scol	Other scol	Age at diagnosis scoliosis			Age at surgery
Yes						21			
			Yes			2			9
	Yes			Yes		22			
			High grade L-S SPL - Surgery			9			12
Yes									
	Yes				Yes				
Yes						22			
			Yes			?12			13
Yes						12			
Yes						27			
	Yes					12			
	Yes					17			

	Growth charts	arts Age	4	5	6	7	8	9	10	11	12	13	14	15
	Percentile normal growth													
								1	1	1	1	1	1	2
				2	2	2	2	2	2	2	2			
			2	2	2.5	3	3	3	3	3	3			
			4		2	2	2	2	2	2	2	2	2	2
					1	1	1	5	20	30	50	45	40	
											3	3	3	10
			2	2	2	1.5	1.5	1	1.5	2	1.5	1.5	2	2
										1	1	1	1	1
										3	2.5	2	1.5	1
											3	3	3	3
			75	50	10	9	4	3	3	2	2.5	3	3	5
			2	2	1.5	2	2	2.5	3	3	9	11		
										3	3	2.5	2.5	
			3	2.5	2.5	2	10	15	25	25	22	26	30	
			3	3	2.5	2.5	3	5	4	2	2.5	2.5	3	9
			2.5	2.5	2.5	3	5	10	9	7	5	5		
			1	2.5	3	8	25	25	25	25				
			2.5	2.5	2	2	2	2	1	1	1	1	1.5	1.5
						2	2	2.5	3	3	3	5	10	10
			1	1	1	1	1	1	1	1	1	1	1	1
			3	3	3	2.5	2.5	2.5	2	2	1	1	1	2
									2.5	2.5	3	3	9	9
					3	2.5	2.5	2	2	2	2	2	2.5	2.5
							2	2	2	2.5	2.5	2.5	2.5	
			3	2.5	2.5	2.5	2	2	2	2	1.5	1	1	2
										2	2	2.5	3	3
					2	2	2	2	2	1.5	1	1	1	
			3	3	3	2.5	2.5	3	3	3	2.5	2	1.5	1.5
								2.5	2	2	2	1.5	2	2.5
				2	1.5	1	10	35	52	50	50	35	30	25
						3	3	3	3	3	5	5	5	5
										3	3	5	6	6
					2	3	2	2	3	5	25	25	5	5
			3	2	2	2.5	3	3	4	5	3	2.5		3
									40	25	10	10	25	45
			1	1	1	1	1	1	1	1	1	1	1	1
			1	1	1	1	1	1	1	1	1	1	1	1

16	17	Actual height	3	4	5	6	7	8	9	10	11	12	13	14
3									113	118	122	127	135	142
				94.5	95.5	100	105	112	115	120	129	135		
			79	92	99	104	111	114	120	125	130	136		
2			56		58									
						98	100	105				155		
25												132	133	134
2.5			84	88	93	98	102	106	109	117	124	128	133	140
1											113	122	126	128
2											130	131	135	138
3												133	139	144
15				104	104	106	108	112	115		121	130	135	140
			83	88	94	99	105	113	117	124	130	137	147	
											128		137	138
			86	92	97	103	108	120	126	132	137	143	151	159
15						103	108	114	120	126	132	137	143	148
				90	98	104	112	117	124	129	133	137	144	
			76	82	96	105	112	121	127	133	136			
1.5								110	112	116	121	128	133	138
5							109	112	118	125	130	135	143	153
1					88	93	99	104	108	114	119	123	126	126
2				92	100	103	107	110	115	117	122	127	131	138
8										124	127	134	141	150
2.52						103	107	111	113	118	125	130	135	143
										117	126	132	137	140
2.5								112	116	123	129	130	138	142
											124	128	139	146
						100	103	108	114	118	122	126	135	139
1			87	91	98	103	108	113	118	125	131	136	139	141
4									118	122	125	132	136	141
					93	97	100	119	130	136	144	150	154	159
5														
5											132	137	145	149
						96	108	109	116	122	134	142	144	144
			88	93	96	101	108	115	120	126	133	139	144	147
50										135	138	140	146	156
1			81	87	93	96	100	103	107	110	114	117	121	126
			78	83	86	91	96	103	110	117	120	127	133	136

**15**	**16**	**17**	**18**	Growth hormone		Dose IU/W	Months 1	Dose IU/W	Months 2	Dose IU/W
						12	15			
145	147	148				24	36	42	48	35
				Saizen	Norditropin	12	14	16	6	20
				Somaton	Saizen	7	31	9	11	14
?73		?75	Data?			8	36	12	18	16
159						7	12			
157	162	165				14	12	30	12	
143	145	147	148			11	12	14	60	
						18	3	nil	24	18
130	136	140	146	Somaton	Saizen	14	3	12	2	28
143	155	158	164			18	72			
153	160	164				20	24	18	6	20
148	155			Somaton	Saizen	14	2	7	4	14
				Genotropi		8	3	9	7	12
						18	15			
						14	12			
				Norditrop		9	31	12	8	15
						16	6	18	18	24
153	153	156		Somaton	Saizen	18	15	24	20	28
				Genotropi		8	23	12	9	18
						9	15	12	18	nil
				Humatro		7	36	12	9	18
141	143	144								
159	161	163		Somaton	Genotropi	10	5	18	40	18
126	127		130			21	3	18	10	
						18	30			
141	144									
156	162	164		Humatrop	Saizen	17	3	18	8	19
145		148		Genotrop		12	14	18	15	20
						12	24			
				Genotrop		24	6	29	6	34
				Genotrop		14	8	7	5	
144	147	149		Humatrop	Saizen	12	16	18	14	30
154				Genotrop		14	11	18	14	16
				Genotrop		16	3	14	3	16
				Genotrop		13	12	14	3	32
143	144			Genotrop		29	6	34	12	36
146	151									
165				Humatrop	Saizen	11	27	14	7	15
				Somatono		24	15	30	27	42
150	151			Pit-hGH	Somaton	4	4	nil	15	12
144						12	48	24	24	
				Humatrop		9	9	12	7	16
168	173	174				16	9	18	42	
131						20	12	30	48	
				Genotrop		22	6	24	12	29
140				Genotrop		7	15	9	18	12

Months 3	Dose IU/W	Months 4	Dose IU/W	Months 5	Dose IU/W	Months 6	Dose IU/W	Months 7
6								
13	28	4	30	14				
12	16	9	18	24	20	4		
54								
9	28	3						
8	nil	18	30	16				
9								
4	17	1	14	24	18	24	35	8
6	14	4	nil	8	14	16	16	12
25	20	5	21	12				
12								
13	32	32	34	8	35	20	40	11
32	nil	16	25	16	29	30		
6	12	12	15	18				
3	12	18	nil	4	12	7		
5	24	18						
6	20	3	21	8	22	10	38	3
12	30	11	nil	1	30	5		
19	36	3	39	7	42	11	48	3
14	3	203	14	2	20	11	28	5
6								
14	26	11						
14	24	4						
18	40	9	46	46	44	3	56	15
6	40	6	44	22	48	2		
15	20	21	25	9	nil	2	30	28
3								
53	18	24						
30	24	24	30	15	32	15	36	3
8	30	3	31	15	32	20		
20	21	7	nil	11	20	22	24	25

Mg/Kg	Mg/Kg	Mg/Kg	Mg/Kg
0.55	0.48		
0.72	0.82	0.44	0.52
0.65	0.55	0.9	0.79
0.77	0.95	0.5	
0.62	0.67	0.38	
0.43	0.45		
0.4	0.9		
0.59	0.67	0.58	
0.35	0.47	0.81	
0.83	0.81		
0.48	0.32		
0.49	0.38		
0.48	0.25	0.57	0.61
0.4	0.33		
0.46	0.44		
0.6	0.41	0.65	0.53
0.91	1.14	0.92	
1	1.05	0.63	0.84
0.6	0.63	0.47	
0.62	0.53	0.69	0.63
0.66	0.37	0.8	0.32
0.5	0.9		
0.93	0.79		
0.5	0.34		
0.39	0.37	0.7	0.64
0.51	0.48	0.99	0.86
0.45	0.55		
1.14	1.3	0.97	1.19
0.72	0.34	0.87	0.95
0.44	0.98		
0.54	0.43	0.67	
0.57	0.5	0.73	
0.52	1.2	0.96	
1.26	0.98		
0.58	0.42	0.71	0.66
0.6	0.8		
0.63	0.38		
0.49	0.23	0.39	
0.67	1.04	0.8	
0.39	0.28		
0.74	0.62	0.94	0.78
1.29	0.88		
0.53	0.58	0.81	0.59
